# PROFILE OF RESPIRATORY PROBLEMS IN PATIENTS PRESENTING TO A REFERRAL PULMONARY CLINIC

**DOI:** 10.4103/0970-2113.44129

**Published:** 2008

**Authors:** Angira Dasgupta, Anirban Bagchi, Saikat Nag, Sujan Bardhan, Parthasarathi Bhattacharyya

**Affiliations:** The Institute of Pulmocare and Research, CB-16, Salt Lake, Sector - I, Kolkata - 700064, India

**Keywords:** OPD Statistics, COPD, Asthma, Cough, Undiagnosed etiology, Hemoptysis, ILD.

## Abstract

Analysis of OPD data of 2012 patients in a referral pulmonary clinic at Kolkata was done following a protocol-based approach. Obstructive airway diseases (COPD and asthma) were the most common (43%) problem followed by infective lung diseases (15%) including tuberculosis, bronchogenic carcinoma (8%), ILD (4%), haemopty-sis of undiagnosed etiology (4.5%), chronic cough of undiagnosed etiology (6.5%) and pleural diseases (4.6%). Other diseases like obstructive sleep apnoea, sarcoid-osis, systemic diseases with lung involvements etc., and non respiratory problems formed the rest (14.4%).

## INTRODUCTION

The burden of respiratory diseases in India is huge. Although some epidemiological data is available on major respiratory problems such as asthma^1^–^3^, tuberculosis^4^, COPD^5^,^6^ and bronchogenic carcinoma^7^,^8^ an efficient database for different respiratory diseases is absent. Here we present the diagnostic profile of 2012 patients presenting to a tertiary respiratory care OPD at Kolkata.

## METHODS

The data has been collected from the records of the Institute of Pulmocare and Research which offers referral OPD services for patients from private practitioners and local health care units. The institute is a well-equipped one as far as human resources are concerned with three consultants and adequate number of ancillary staffs. It caters mostly to an urban population from the economically middle class background.

**Fig 1:**
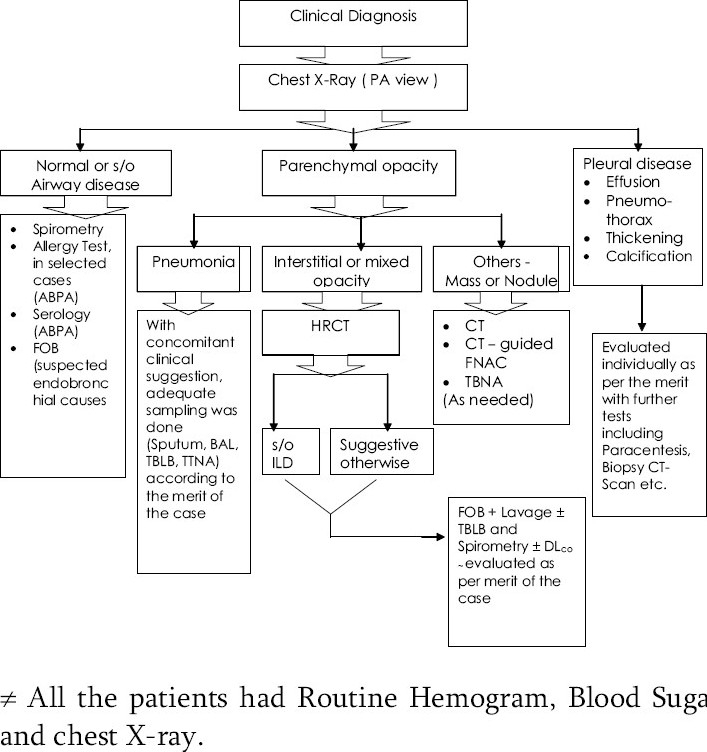
The Protocol ≠ All the patients had Routine Hemogram, Blood Sugar and chest X-ray.

The institute uses a protocol-based approach for evaluation and diagnosis of patients presenting with respiratory problems. At the initial visit, after allotting a registration number, a thorough history is taken and clinical examination performed. Depending upon the clinical impression the patients are evaluated in accordance to a protocol as far as possible (figure 1).

2012 consecutive patients who attended the clinic between March 2006 and October 2006 were incorporated for analysis from the record of the patients. The best possible effort was made to reach a final diagnosis in each of them. Although most of the patients were investigated on a day care basis, whenever necessary, we admitted them for the sake of completing the evaluation. This included sick patients with active infection or exacerbation of airway diseases. Final diagnosis was made taking into consideration both the clinical and investigation results. In most of the cases, diagnoses were limited to broad diseases (e.g. ILD) for the purpose of analysis. Further subgroups analyses were not attempted since appropriate tests (collagen profiles, open lung biopsy etc.) could not be performed in all cases for logistic reasons.

The criteria for diagnosis of different diseases were as follows:

Asthma and **COPD** were diagnosed as per the GINA^9^ and GOLD^10^ guidelines respectively using the history and presenting symptoms in combination with spirometry.

Infective problems were diagnosed with the evidence of purulent expectoration with systemic features of infection as fever, myalgia etc. along with and radiological evidence of alveolar opacities and/ or presence of leucocytosis in the peripheral blood.

Pleural diseases were diagnosed with the help of chest x-rays, pleural fluid analysis, CT scan and pleural biopsy as per the requirement of the cases.

Interstitial Lung diseases were diagnosed clinicoradiologically using the ATS-ERS^11^ guidelines as far as possible. We took the help of HRCT scan of the thorax in all cases and bronchoscopy with transbronchial lung biopsy or bronchoalveolar lavage or open lung biopsies were done wherever possible. However, financial and logistic problems did not make it possible to complete the workup in all cases. Hence it was not possible to appoint specific etiologies or further subclassify the idiopathic ILD group.

Bronchogenic carcinoma was diagnosed after biopsy or CT guided fine needle aspiration cytology of lung mass or accessible lymph nodes, from histopathological examination bronchial biopsy or from cytological examination of transbronchial needle aspiration smear.

Obstructive sleep apnea was diagnosed when there were more than 10 spells of hypopnea/apnea per hour in a polysomnography with an intact respiratory effort^12^.

Sarcoidosis was diagnoses by histological demonstration of noncaseating granuloma from at least two tissue sites in the background of typical or suspected clinical and radiological findings^13^.

Tuberculosis was diagnosed by sputum for AFB and culture for mycobacterium tuberculosis and atypical mycobacterial infection.

In cases where an etiological diagnosis could not be reached due to a lack of adequate evaluation mostly due to financial constraints, a symptomatic diagnosis (haemoptysis of undiagnosed etiology, shortness of breath? cause / undiagnosed etiology, lymphadenopathy of undiagnosed etiology and cough? cause/ undiagnosed etiology, chest pain of undiagnosed etiology etc.) were made.

Due to financial and other constraints, adequate evaluation was not possible in a good number of patients of haemoptysis and shortness of breath. In such cases a symptomatic diagnosis as mentioned above was kept. Finally, the data obtained was analyzed.

## RESULTS

A total of 2012 patients were included, of them 12 were lost o follow-up, and the remaining 2000 were finally evaluated. The Male: Female ratio was 2:1. The relative prevalence of different diseases was as given in table 1.

**Table 1:** Prevalence of the different diseases

Disease	Number of patients (n=2012)	% of all cases
Asthma	534	26.54
COPD	245	12.18
Infective problems	144	7.16
Soft tissue mass (proved bronchogenic carcinoma)	78(59)	3.88
TB (typical and atypical)	146	7.26
Pleural pathology	56	2.78
Obstructive Sleep Apnoea	39	1.94
Interstitial Lung Disease	87	4.32
Sarcoidosis	24	1.19
Cough of undiagnosed etiology	101	5.02
SOB of undiagnosed etiology	80	3.97
Hemoptysis of undiagnosed etiology	88	4.37
Systemic diseases with lung involvement	9	0.45
Pain chest of undiagnosed etiology	17	0.84
Lymphadenopathy of undiagnosed	14	0.69
etiology		
Others	41	2.04
Other not enlisted problems and	297	14.76
non resp problems		


## DISCUSSION

This is an OPD-based analysis of data. Although much inferior to a proper epidemiological survey, it still carries significant importance since there is a dearth of such data in our country. In our observation, asthma (26.54%) has the highest prevalence, followed by COPD (12.18%). Tuberculosis (7.26%) and other infective problems (7.16%) such as pneumonia and bronchiectasis come next in frequency. The data shows that the obstructive airway problems (primarily asthma and COPD) form a good bulk (38.72%) even in referral respiratory OPD practice. Most of these patients were being treated elsewhere without satisfactory improvement. A systematic analysis of them could be helpful to appreciate the reasons for inadequate control or dissatisfaction. In our experience, the airway diseases could also include some bronchiectasis patients without active infection while some patients with infective exacerbations of bronchiectasis could have been incorporated in the category of infective diseases in short of proper evaluation with HRCT chest. Infective problems including tuberculosis consisted of 14.42% of the total number of cases. Tuberculosis per se actually comprised 7.26% of the total cases. All the patients presenting with pulmonary soft tissue mass could not be clearly evaluated due to financial constraints. Definitive malignancy could be detected in 59 out of 78 and it is likely that in most of the remaining cases malignancy was the probable diagnosis.

A good number (163) of patients presented with haemoptysis. But, the final etiological diagnosis could not be offered to all for want of evaluation mostly because of financial and other constraints. In 24 of these patients a diagnosis of tuberculosis was made. Evidence of bronchiectasis on X-Ray or HRCT chest was detected in 41 subjects. In addition, 10 cases had bronchogenic carcinoma and in the remaining patients (88), the diagnosis was uncertain.

The other problems like ILD (4.32%) and pleural diseases (2.78%) were not uncommon and we diagnosed about 24 (1.19%) patients of Sarcoidosis in the course of evaluation.

Of the patients who were lost to follow-up 4 had cough, 2 had shortness of breath and the remaining 6 who had nonspecific symptoms have been included in the category of other not enlisted problems /non respiratory problems group for the purpose of analysis.

Though there are several OPD/ indoor-based statistics, they mostly concern the profile of presentation or some other aspects of a particular problem. In one CDC report, asthmatics comprised 1.5% of the total OPD patients^1^. One other study on the local prevalence of asthma had reported the mean prevalence to be 2.38%^2^,^3^. In India, too, the prevalence of asthma has been recorded to be less than 5 % in most studies^3^. However, we have no data reporting the local prevalence in eastern India. Community based data regarding COPD is even scantier^5^,^6^. The prevalence of COPD in Punjab appeared to be close to 5%^5^,^6^. The different prevalence in different parts of India could be explained by the varied tobacco smoking habits in different parts of the country. Similarly, reports regarding lung cancer prevalence are mostly hospital based^7^–^8^,^14^–^17^ and hence not comparable to an OPD based data, although lung cancer formed the most common malignancy in males in six of ten centers evaluated by the ICMR^18^.

The attendance in a referral OPD depends on different variables like proximity to patients' residence, available facilities, awareness of the referring authority etc. Hence, OPD based data may have some limitations. However, a systematic multicentric survey on a protocol based OPD data – may give some more insight into the regional variations in the prevalence of different diseases in the different parts the country. Repeated and periodic evaluation with the same protocol may also give an idea about the trend of the diseases and success of different intervention measures.

This study certainly has some obvious limitations. Most of the cases were referred cases. So probably, the patient profile does not truly reflect any particular disease prevalence of the representative population. The protocol followed has not been used in any previous study. Hence its appropriateness remains to be validated. We have also not been able to follow the protocol strictly due to financial or other constraints in some of the cases. Pneumonia and infective exacerbations of bronchiectasis were clubbed in the same group. Subgroup analyses could not be made due to logistic problems in several conditions such as interstitial lung disease.

Despite these limitations our study highlights the spectrum of the diseases presenting to a tertiary clinic in Eastern India when there is a dearth of even similar data in literature. Though it lacks the importance of an epidemiological survey, it still merits importance since (1) it gives an overall idea about the prevailing chest problems in a community, (2) the status at presentation signifies the level of awareness of the patients and/or the referring doctors, (3) it gives impetus for further in-depth analysis that may be worthwhile for inadequate control of certain problems in the community, and finally (4) it helps to compare similar, if any, statistics from different parts of the country.
